# GC-based chemoprofile of lipophilic compounds in Altaian *Ganoderma lucidum* sample

**DOI:** 10.1016/j.dib.2018.03.098

**Published:** 2018-03-26

**Authors:** Oleg B. Shevelev, Alisa A. Seryapina, Evgenii L. Zavjalov, Lyudmila A. Gerlinskaya, Tatiana N. Goryachkovskaya, Nikolay M. Slynko, Leonid V. Kuibida, Sergey E. Peltek, Arcady L. Markel, Mikhail P. Moshkin

**Affiliations:** a Institute of Cytology and Genetics, Siberian Branch, Russian Academy of Sciences, 10 Prospekt Lavrentyeva, Novosibirsk 630090, Russia; b Novosibirsk State University, 2 Pirogova Str., Novosibirsk 630090, Russia; c Voevodsky Institute of Chemical Kinetics and Combustion, Siberian Branch, Russian Academy of Sciences, 3 Institutskaya str., Novosibirsk 630090, Russia; d Tomsk State University, 36 Lenina Avenue, Tomsk 634050, Russia

## Abstract

The presented data contains information about component composition of lipophilic compounds in *Ganoderma lucidum* fungal body sample obtained using gas chromatography and subsequent mass spectrometry.

**Specifications Table**

**Table:** 

Subject area	Chemistry
More specific subject area	Gas chromatography-based chemoprofile of fungal body of *Ganoderma lucidum*
Type of data	Table and figure
How data was acquired	Gas chromatography and mass spectrometry; Agilent Technologies 6890N chromatograph with a quartz capillary column DB-1 and an Agilent Technologies 5973N quadrupole mass spectrometer.
Data format	Analyzed
Experimental factors	Mushrooms were pre-air-dried to constant weight and grinded using a jet mill to a very fine powder, shredded mushrooms were dried in an air thermostat at a temperature of 70 °C to a constant weight.
Experimental features	To obtain the fingerprint of the lipophilic compounds of *Ganoderma lucidum*, 1.0 g of air-dry mass of the crushed fruit body of the fungus was boiled with 10 ml of methyl alcohol for 1 h, the extract was filtered and the filtrate was passed through a column with an Al2O3 layer 15 mm in height for dehydration, 1 ml was taken for analysis by gas chromatography.
Data source location	Novosibirsk, Russia
Data accessibility	Data is with this article.
Related research article	Hypotensive and Neurometabolic Effects of Intragastric Reishi (*Ganoderma lucidum*) Administration in Hypertensive ISIAH Rat Strain (PHYMED-D-16-01100R1) [3]

**Value of the data**

● The data could be potentially valuable to the scientific community, because it is new data about component composition of *Ganoderma lucidum* from Altai region.
● It could be compared to the data of the same fungus from another regions.
● The data is needed to investigate impact of individual compounds to organism.

## 1 Data

See Fig. S1 and Table S1.

## 2 Experimental design, materials, and methods

To obtain the fingerprint of the lipophilic compounds of *Ganoderma lucidum*, 1.0 g of air-dry mass of the crushed fruit body of the fungus was boiled with 10 ml of methyl alcohol for 1 h, the extract was filtered and the filtrate was passed through a column with an Al_2_O_3_ layer 15 mm in height for dehydration, 1 ml was taken for analysis by gas chromatography. The analysis was carried out on an Agilent Technologies 6890N chromatograph with a quartz capillary column DB-1 and an Agilent Technologies 5973N quadrupole mass spectrometer. Chromatographic mass spectrometric analysis of the samples was carried out both to measure the total ion current in the scanning regime in the mass range 10–800 amu, and in the mode of selective ion scanning.

The identification of components present in the methanolic extract was based on direct comparison of the retention indices and mass spectral data with those for standard compounds, and by computer matching with the Wiley 229, Nist 107, Nist 21 Library, as well as by comparison of the fragmentation patterns of the mass spectra with those reported in the literature [2]. The Kovacs retention indices were calculated from the retention times of Lucero et al. [1].

## Appendix A Supplementary material

Fig. S1. Gas chromatogram of lipophilic substances of the *Ganoderma lucidum* fungal body sample.

Table S1. Fingerprint of lipophilic compounds in *Ganoderma lucidum* fungal body sample.
