# Supplementary material for: GC-based chemoprofile of lipophilic compounds in Altaian Ganoderma lucidum sample
Source: Data Brief. 2018 Mar 26;18:1054–6. doi: 10.1016/j.dib.2018.03.098 (PMC5996614; doi:10.1016/j.dib.2018.03.098)
Supplement: Supplementary file 1 — Supplementary material [file mmc3.docx]

| Name of compound | RT (min) | RI | RI_lit_ | Peak Area (%) |
| --- | --- | --- | --- | --- |
| 1-tetradecene | 9,192 | 1424 | 1412 | 4,458 |
| 2,4-di-tert-butylphenol* | 9,616 | 1490 | 1510 | 0,206 |
| Cetene | 9,982 | 1586 | 1590 | 0,422 |
| Methyl pentadecanoate | 11,011 | 1815 | 1811 | 0,368 |
| Pentadecanoic acid | 11,249 | 1872 | 1865 | 0,587 |
| Palmitic acid | 11,604 | 1950 | 1961 | 7,985 |
| 1-eicosene | 11,852 | 2002 | 1997 | 0,477 |
| Phytol | 12,208 | 2070 | 2080 | 1,607 |
| Nonadecane-2-one | 12,291 | 2098 | - | 1,278 |
| Oleic acid | 12,782 | 2167 | 2171 | 26,254 |
| Stearic acid | 12,897 | 2185 | 2169 | 4,015 |
| Unidentified substance | 13,092 | 2214 | - | 0,230 |
| Unidentified substance | 13,145 | 2221 |  | 0,983 |
| 11,14-Eicosadienoic acid, methyl ester | 13,453 | 2262 | 2279 | 1,193 |
| Eicosenoic acid, methyl ester | 13,541 | 2274 | 2279 | 1,443 |
| Methyl arachidate | 13,814 | 2308 | 2297 | 2,867 |
| Eicosan-1-ol | 13,872 | 2315 | 2275 | 0,179 |
| Eicosenoic acid | 14,035 | 2333 | - | 3,177 |
| Eicosanoic acid | 14,246 | 2356 | 2365 | 1,685 |
| Erucic acid | 14,405 | 2373 | - | 1,377 |
| Pimaric acid | 14,810 | 2413 | - | 0,874 |
| Callitrisic acid | 15,030 | 2433 | - | 1,234 |
| Heicosanoic acid | 15,250 | 2452 | 2463 | 0,786 |
| Abietic acid | 15,594 | 2482 | 2512 | 0,351 |
| Dehydroabietic acid | 15,990 | 2513 | - | 0,919 |
| Phthalic acid, bis(6-methylheptyl) ester* | 16,078 | 2519 | 2545 | 0,455 |
| cis-11,14-eicosadienoic acid | 16,202 | 2527 | - | 0,382 |
| Docosanic acid | 16,580 | 2553 | 2567 | 2,068 |
| Unidentified substance | 16,977 | 2580 |  | 1,204 |
| Hexacosane | 17,188 | 2593 | 2600 | 0,191 |
| Tricosenoic acid | 17,761 | 2627 | - | 0,327 |
| Tricosanoic acid | 18,236 | 2653 | 2668 | 0,958 |
| 6-Nonadecyltetrahydro-2H-pyran-2-one | 19,769 | 2727 | - | 0,614 |
| Tetracosanoic acid | 20,403 | 2752 |  | 0,892 |
| Unidentified substance | 20,536 | 2758 |  | 0,170 |
| squalene | 22,024 | 2813 | 2817 | 1,599 |
| (22E)-3,5-Cycloergosta-7,9(11),22-trien-6-ol | 22,905 | 2843 | - | 1,008 |
| Stigmasterol | 24,966 | 2905 | 2900 | 0,959 |
| Anthraergostapentene | 25,979 | 2929 | 3020 | 8,976 |
| α-Spinasterone | 26,376 | 2938 | - | 0,943 |
| 3-hydroxy-4,4,10,13-tetramethyl-7-oxo-2.4.7.8.9.10.11.12.13.14.15.16.17-tetradecahydroacetic acid | 27,407 | 2961 |  | 1,143 |
| Anthiaergosta-5,7,9,14-tetraene | 28,014 | 2975 |  | 1,459 |
| Unidentified substance | 30,278 | 3021 | - | 0,465 |
| Vitamine E | 33,220 | 3077 |  | 0,222 |
| Dehydroergosterol | 33,907 | 3089 |  | 1,649 |
| Ergosterol | 36,030 | 3124 | 3123 | 7,170 |
| (3β,5α,22E)-Ergosta-14,22-dien-3-ol | 37,088 | 3140 |  | 5,968 |
| 7,22-Ergostadienone | 38,224 | 3157 |  | 4,000 |
| β-sitosterol | 40,972 | 3196 | 3201 | 5,503 |
| 24-Methylenecholesterol | 42,311 | 3213 |  | 2,991 |
| Anthraergostatetraenol | 46,513 | 3262 |  | 1,038 |
| Ergosta-4,6,8(14),22-tetraen-3-one | 46,918 | 3267 |  | 1,677 |
| Unidentified substance | 50,548 | 3305 |  | 1,175 |
| β-sitostenone | 53,780 | 3336 |  | 0,322 |

* Alleged anthropogenic impurity
