# Supplementary material for: GC-based chemoprofile of lipophilic compounds in Altaian Ganoderma lucidum sample
Source: Data Brief. 2018 Mar 26;18:1054–6. doi: 10.1016/j.dib.2018.03.098 (PMC5996614; doi:10.1016/j.dib.2018.03.098)
Supplement: Supplementary file 2 — Fig. S1. Gas chromatogram of lipophilic substances of the Ganoderma lucidum fungal body sample. [file mmc1.docx]

Conflict of Interest

There is no conflict of interest.
