# Supplementary figures and images for: GC-based chemoprofile of lipophilic compounds in Altaian Ganoderma lucidum sample
Source: Data Brief. 2018 Mar 26;18:1054–6. doi: 10.1016/j.dib.2018.03.098 (PMC5996614; doi:10.1016/j.dib.2018.03.098)

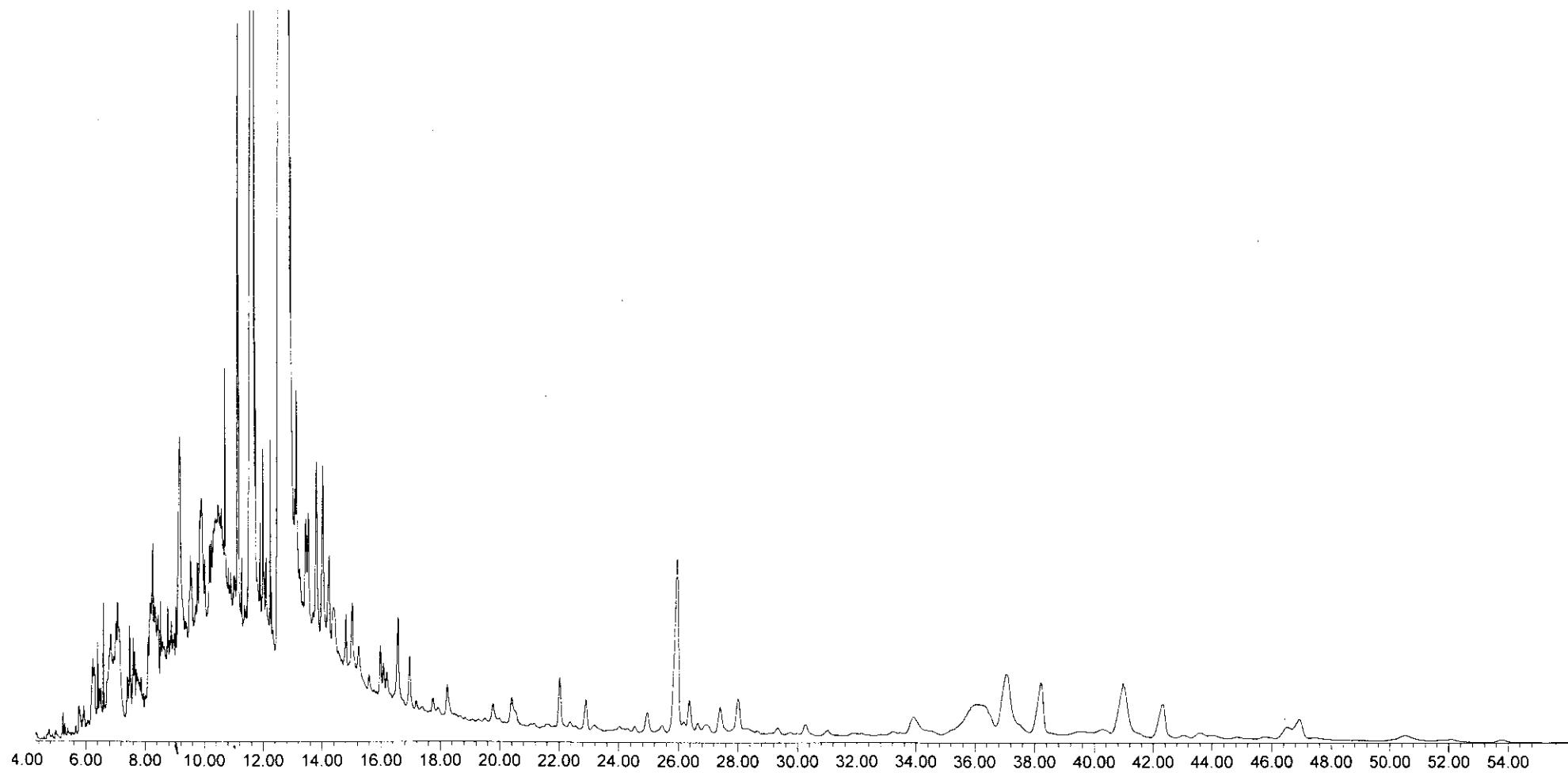

Supplement: Supplementary file 3 — Table S1. Fingerprint of lipophilic compounds in Ganoderma lucidum fungal body sample. [file mmc2.pdf]
